# An artificial neural networks approach for assessment treatment response in oncological patients using PET/CT images

**DOI:** 10.1186/s12880-017-0181-0

**Published:** 2017-02-13

**Authors:** Mariana A. Nogueira, Pedro H. Abreu, Pedro Martins, Penousal Machado, Hugo Duarte, João Santos

**Affiliations:** 1 CISUC - Department of Informatics Engineering - University of Coimbra, - Pólo II Pinhal de Marrocos, Coimbra, 3030-290 Portugal; 2 IPO-Porto Research Centre (CI-IPOP), Rua Dr. António Bernardino de Almeida, Porto, 4200-072 Portugal

**Keywords:** Artificial neural networks, Images descriptors, PET/CT images, Treatment response assessment

## Abstract

**Background:**

Positron Emission Tomography – Computed Tomography (PET/CT) imaging is the basis for the evaluation of response-to-treatment of several oncological diseases. In practice, such evaluation is manually performed by specialists, which is rather complex and time-consuming. Evaluation measures have been proposed, but with questionable reliability. The usage of before and after-treatment image descriptors of the lesions for treatment response evaluation is still a territory to be explored.

**Methods:**

In this project, Artificial Neural Network approaches were implemented to automatically assess treatment response of patients suffering from neuroendocrine tumors and Hodgkyn lymphoma, based on image features extracted from PET/CT.

**Results:**

The results show that the considered set of features allows for the achievement of very high classification performances, especially when data is properly balanced.

**Conclusions:**

After synthetic data generation and PCA-based dimensionality reduction to only two components, LVQNN assured classification accuracies of 100%, 100%, 96.3% and 100% regarding the 4 response-to-treatment classes.

## Background

In an oncological context, Positron Emission Tomography – Computed Tomography (PET/CT) became a standard not only in disease staging but also as a quantitative method to monitor treatment response and, in recent years, in the early prediction of final response when only few cycles of the programmed treatments have been performed [1].

Basically, the images obtained through PET/CT show the metabolism of a specific radiotracer like 18F-FDG (18F-fuoro-deoxyglucose) or 68Ga-DOTANOC (68Ga-DOTA-Nal-Octreotide), throughout the body. As cancer cells are highly proliferative ones, they take a lot of the radiotracer and will appear as intense activity areas. However, some other causes can produce such activity like infections, inflammations or muscle activity, which turn the analysis of PET/CT images into a complex process.

One of the most widely used measures to quantify the uptake of radiotracers is the Standardized Uptake Value (SUV) [2]. Its calculation takes into account the differences between normalized values of body weight, lean body mass, surface area, and injection dose. However, multiple factors can influence the SUV calculation [3]. These factors can be related to/ influenced by:

1. Imaging Physics – The function of PET is to form an image of the spatially varying concentration of positron-emitters. However, the image resolution is typically on the order of 4 to 7 mm full-width half-maximum leading to partial volume effects or partial volume errors [4], which can bias the true concentration value for small objects in the image;
2. Patient status – The status of the patient can directly influence the SUV value. Factors like glucose and insulin levels in the blood can increase FDG uptake by muscles with less available molecules for equilibration in tumor tissue. Also chemotherapy treatments can result in impaired renal function, reducing the clearance of plasma FDG through the kidney that will increase the SUV value in comparison to the initial PET scan;
3. Dose extravasation – Dose extravasation, defined as the efflux of radiotracer from a vessel into surrounding tissues during intravenous infusion, results in an underestimated SUV, as the activity that effectively enters the circulation and distributes through the organs is inferior to the activity used for SUV calculation;
4. Scan Protocol – There are some components encompassing the scan protocol that can sway the SUV value [5]. These components are: (1) the uptake time: interval between injection and image acquisition; (2) measuring the residual activity in the syringe; (3) accurate measurement of patient weight; (4) synchronization of clocks used for dose assays and scanning; (5) patient respiratory motion; (6) correct data entry;
5. Data Processing – Two steps compose Data Processing: corrections for confounding effects like attenuation, scattered and random coincidences, scanner deadtime, and detector efficiency variations; and image reconstruction where the raw data is transformed into an image of relative radioactivity concentration;
6. Scanner Calibration – The scanner calibration comprises two phases: estimation of scanner calibration factor and conversion of the image unit to SUVs. An erroneous value inserted in the calibration factor can originate an error in calibration and in SUV calculation.

Throughout the years, some other metrics derived from SUV have emerged to evaluate treatment response:

1. SUVmean and Region of Interest (ROI) definition – This measure consists in the average of SUV value in a tumor ROI. However, some issues arise mainly with respect to image noise and limited image resolution of PET. Another issue is related to the generalization of the use of this measure (known as reproducibility). This point is strictly related to the tumor ROI identification, which depends on the physician preferences related to margin drawings;
2. SUVmax – The idea behind the computation of SUVmax is to identify the maximum SUV value in a single voxel within a tumor ROI. As this measure describes a value of a lesion based on a single voxel, it eliminates the issue presented by SUVmean and ROI (maximum value is invariant to small variations of ROI). However, this measure can be considered more biased and noisy in comparison to other SUV variations, as it is substantially affected by the reconstruction algorithm. Yet, some studies proved that this bias and noise are less than previously thought [6] and because of that it is the most used metric to evaluate treatment response.

This project is part of a wider study whose goal is to create a new accurate assessment measure of lesion malignancy, that would outperform SUV metrics. In this phase, the goal was to automatically assess the treatment response of oncological patients based on PET/CT images, using an artificial neural network approach, and building proof that the set of features used in this study (based on local image descriptors and clinical information) are capable of characterizing this oncological context. To achieve this, before and after-treatment corrected- and non-attenuation-corrected PET images and CT images from 63 patients suffering from one of two oncological diseases – Hodgkyn disease or neuroendocrine tumors – were collected at IPO Porto center (a tertiary cancer center in Northern Portugal).

Four standard artificial neural network (ANN) architectures were explored – multilayer perceptron (MLP), radial basis function neural network (RBFNN), probabilistic neural network (PNN) and learning vector quantization neural network (LVQNN). These ANN receive an input vector with more than 300 features combining clinical and image descriptor information. For comparison purposes, a baseline classifier was also used – k Nearest Neighbors (kNN).

The results show that the considered set of features allows for the achievement of very high classification performances, especially when data is properly balanced. After synthetic data generation and PCA-based dimensionality reduction to only two components, LVQNN assured classification accuracies of 100%, 100%, 96.3% and 100% regarding the 4 response-to-treatment classes.

Although image descriptors are a common tool to perform medical image analysis, regardless of the goal (e.g., diagnosis, treatment), image modality or anatomy, the complementary information provided by different descriptors or their combination with other clinical data is sometimes overlooked. While some works have illustrated the advantages of such combinations (see [7]), there are many scenarios that would certainly benefit from such strategy. To the best of our knowledge, an assessment of oncological treatment response based on the combination of local descriptors and clinical information from before and after treatment, such as the one presented herein, is entirely novel.

Below, we present an overview of works related to the application of ANN approaches in medical image analysis and also a brief description of the use of image descriptors in medical image context.

### Artificial neural networks approaches

ANN have been widely used in automated medical diagnosis. In particular, feedforward neural networks tend to be the most used ANN. Within the group of feedforward networks, a few particular architectures are usually adopted, such as the multilayer perceptron (MLP), the radial basis function neural network (RBFNN), a variation of the RBFNN known as probabilistic neural network (PNN) and the learning vector quantization neural network (LVQNN). Despite the predominance of these ’standard’ networks, a number of different neural networks architectures is found in literature, resulting from modifications and adaptations to particular problems. Regarding automated diagnosis applications specifically based on image, the most frequently adopted type of network, within the group of standard architectures, is, by far, the MLP, followed by RBFNN. PNN has also been used in several studies. Other network models have been specifically designed to operate directly on images, such as Convolutional Neural Networks and Massive Training Artificial Neural Networks [8, 9]. This subsection addresses some relevant examples.

Verma and Zakos [10] used a BP (back propagation)-MLP model with one 10-unit hidden layer for discrimination between benign and malignant breast microcalcifications. With only three inputs – two gray-level features and the number of pixels –, computed from microcalcification regions in mammograms, an accuracy of 88.9% was achieved.

Halkiotis et al. [11] proposed a method to detect cluster breast microcalcifications. Five features were computed from the candidate ROIs, four of them being moments computed from the gray-level histogram and the remaining being the number of objects in a limited neighborhood. These features were input to a BP-MLP with a 10-unit hidden layer for classification as either clustered microcalcification or not. A sensitivity of 94.7% was obtained, with 0.27 false findings per image.

Papadopoulos et al. [12] developed a system also for the detection of microcalcification clusters. From mammogram ROIs, 22 intensity, shape and texture cluster features were extracted. Features of candidates that passed a rule system aiming at false positive elimination were subjected to principal component analysis for posterior dimensionality reduction. Dimensionality was reduced to 9, through the elimination of components that contributed with less than 3% of the overall data variance. Then, the 9 features were fed to a two-hidden-layer BP-MLP, the first layer having 20 units and the second having 10. Area Under the Curve (AUC) values of 0.91 and 0.92 were obtained for two different datasets.

Christoyianni et al. [13] used an RBFNN classifier, first for detecting abnormal breast tissue and, secondly, to discriminate between benign and malignant breast lesions. Three feature vectors were compared: one consisted of gray-level histogram moments, a second one was composed of statistics computed from the co-occurrence matrix and the third onde was composed of the principal components (5 in the case of abnormality detection and 8 in the case of discrimination between benign and malignant lesions) of the coefficients resulting from independent component analysis (ICA). The ICA-based feature vector was the highest performing one in both classification tasks, with an accuracy of 88.23% in the abnormality detection scenario and of 79.31% in the benign from malignant discrimination.

Chen et al. [14] used a PNN for discrimination between two types of liver tumors (hemageoma and hepatoma), based on three image features computed from CT ROIs - correlation, sum entropy and normalized fractional Brownian shape. An accuracy of 83% was obtained.

In some medical image-based diagnosis studies, network models are designed to operate directly on images. Examples are Convolutional Neural Networks – used, for instance, in [15] for detection of abnormal breast tissue in mammogram ROIs, achieving an AUC of 0.87 – and Massive Training Artificial Neural Networks – applied to lung nodule detection and false positive reduction, maintaining a sensitivity of 96.4% and achieving a false positive rate reduction of 33% when compared to a previous system designed by the same authors [16].

Pruning algorithms can be used during training for the elimination of irrelevant network connections performance-wise (e.g. input selection). Setiono [17] applied a pruning algorithm to an MLP in a breast cancer diagnosis application.

Zhou et al. [18] used an ANN ensemble –, i.e. a combination of ANN where final output of the system is a function of the outputs of different ANN – for lung cancer detection.

In [19], Liu presents a study with mammograms to illustrate how ANN can be trained, tested, and evaluated. The main goal of this work is to understand how a radiologist should use the outputs provided by an ANN model as second opinion in computer aided diagnosis.

### Image descriptors

The design of algorithms aimed at providing a description of image parts is a prolific and central research topic in the fields of computer vision and image analysis. Popular local descriptors such as scale invariant transform (SIFT) [20], shape contexts [21], or local binary patterns (LBP) [22] have been used in a wide range of applications such as matching, object recognition and face detection. Image descriptors have also been widely and successfully used in medical image analysis [23]. They appear as an effective tool to assist in the interpretation of different medical image modalities, contributing to improve the diagnosis and monitoring of a large number of diseases. The literature reporting the use of local descriptors in medical image analysis is vast to be comprehensively reviewed and discussed herein, although some relevant and representative works will be presented.

A number of works uses texture descriptors such as LBP in diagnosis and monitoring. For instance, Oliver et al. [24] represented salient micro-patterns using LBP for mammographic mass detection. Dettori and Semler [25] compared the performance of wavelet, ridgelet, and curvelet-based texture descriptors in tissue classification using CT scans. Virmani et al. [26] developed a system to characterize liver ultra sound images using a combination of texture descriptors like the mean, standard deviation, and energy of wavelet coefficients from different families (e.g., Haar or Daubechies). Morgado et al. [27] presented a comparative study of feature extraction and selection on PET images for automated diagnosis Alzheimer’s disease as well as Mild Cognitive Impairment, which is a syndrome associated with a pre-clinical stage of Alzheimer’s disease. In this work, several variants of LBP were analyzed, including a 3D one.

Reddy et al. [28] proposed a confidence-guided segmentation on MRI. The authors used groups of multi-parametric MRI data from different subjects. Each group consisted of a pre-contrast T1-weighted (T1-pre), a post-contrast T1-weighted (T1-post), a T2-weighted (T2) and an attenuated inversion recovery (FLAIR) MRI image. A mask for the enhanced region was generated with the difference T1-pre and T1-post. Then, Mean Intensity, LBP and HOG descriptors were computed for each pixel within the enhanced region mask from each of T1-pre, T1-post, T2 and FLAIR images, and concatenated to form a single feature vector. After that, the feature vectors were fed into a classifier for tumor pixel classification.

Moura and López [7] presented a study in which image descriptors are used individually or combined with clinical data to train classifiers for breast cancer diagnosis. Segmented regions corresponding either to benign lesions or to malignant ones were considered in this study. Besides the mammographic images, other clinical data was collected, such as patient’s age or breast density. The authors selected 11 descriptors that had been successfully used in breast cancer diagnosis and categorized them into four groups: intensity – including intensity statistics, histogram measures, Zernike moments and the Hu set of invariant moments, texture – Haralick features and sets of features computed from the gray-level-run-length and the gray-level-difference matrices, multi-scale texture – Gabor filter banks, wavelets and curvelets and spatial distribution of the gradient – HOG. Within the last group, a novel descriptor was proposed, histograms of gradient divergence (HGD), which is specially designed for round-shaped objects, such as masses, and aims at describing the regularity of their shape.

### Remarks

There is very little documentation on the usage of neural networks for PET image analysis. Furthermore, neural networks in the field of medical imaging are usually employed in registration, segmentation, edge detection, detection and diagnosis of pathologies, and simulation [29], and have not been used for treatment response classification based on before- and after-treatment image features. In addition, the potential advantage in combining the information of different descriptors or combining descriptor information with other clinical information is sometimes overlooked. This study intends to explore such combinations.

### Document outline

The remainder of this paper is organized as follows: Section Methods outlines the methodological steps used in this project concerning the four project phases: Data collection, Data Preprocessing and Treatment Assessment. Section Results and discussion reports the collected results and, finally, Section Conclusions presents the conclusions and proposals for further studies.

## Methods

The development of this project follows a traditional KDD (Knowledge Discovery in Databases) approach [30]. The following subsections provide a complete description of all the phases.

### Data collection

**Fig. 1:**
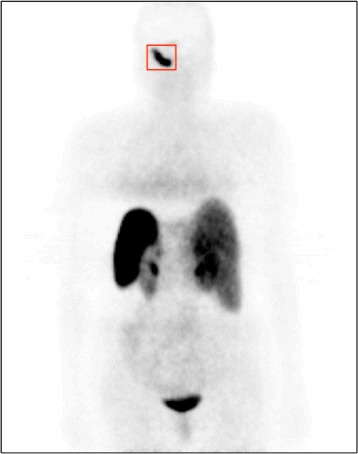
Example of a coronal slice of an attenuation-corrected PET image of a patient suffering from paraganglioma, a neuroendocrine tumor that affects head and neck (head in this particular case)

Data was collected at IPO-Porto by a medical team composed of one nuclear medicine specialist and two physicians. Clinical data from 63 patients, suffering from one of two oncological diseases – neuroendocrine tumors (34) and Hodgkyn disease (29) – was provided (Fig. 1 depicts an attenuation-corrected PET image). Patient clinical data included: pre- and post-treatment PET/CT exams, patient age and tumor stage by the time of the first PET and CT exams and patient weight before and after treatment. In addition, the medical team provided the values of the maximum SUV within the main lesion, the maximum SUV within a reference organ (spleen in the case of neuroendocrine pathology and liver in Hodgkyn disease) and the information of what type of treatment response was observed in each patient. Cases were divided into four classes of treatment response – negative (malignancy increased), neutral (no response), positive incomplete (malignancy decreased but lesion did not disappear) and positive complete (the lesion disappeared). The number of samples per class is: 2 samples of the negative class, 6 samples of the neutral class, 27 samples of the positive (partial) class and 28 samples of the positive (complete) class. In summary, each patient has 8 clinical variables and two PET/CT exams associated. The 8 clinical parameters were combined with before- and after-treatment image features of the lesions computed from the PET and CT images (corrected- and non-attenuation-corrected PET images and CT images).

Before feature extraction, the images were normalized to [0,1] intensity interval, and lesions were segmented using a classical segmentation approach – 2D region growing method with a fixed threshold empirically set to 40%. From each segmented region, several image descriptors were computed and a set of image features were extracted from their outputs. Six texture descriptors with solid reputations in similar applications were selected. Table 1 presents details on descriptor computation and feature extraction.

**Table 1:** Parameters used for descriptor computation and number of features extracted from their outputs – N/A – not applicable

Descriptor	Parameters		#Extracted features
Gray-level histogram	N/A		4
GLCM	directions={0,45,90,135}		22
	distance = 1px		
GLRL	directions={0,45,90,135}		11
Wavelets	#levels=2		28
	type=Daubechies4		
Gabor filters	#scales=3		48
	#orientations=4		
LBP	*R*=8		58
	block size= size of ROI		
			*TOTAL*=171

Thus, 171 image features were computed for characterizing each lesion. For each patient, a 350-sized feature vector was assembled, combining the 8 clinical variables and the 171 ×2 pre- and post-treatment image features of the main lesion. The final dataset is then composed of 63 instances of 350 features.

### Data preprocessing

One is fast to notice two potentially problematic aspects of the dataset: high dimensionality and high imbalancement. In order to assess the effects of these characteristics in the results, experiments were also run after applying PCA for dimensionality reduction (reduction to two principal components, since these preserved over 99% of total data variance) and SMOTE (Synthetic Minority Over-sampling Technique) [31] for synthetic data generation (6 synthetic samples of each of the two most underrepresented classes were generated. This number was chosen based on the results of a set of preliminary experiments).

### Treatment assessment

For classification, ANNs were selected based on their solid reputation with regard to classification tasks. A few standard architectures – MLP, LVQNN, PNN and RBFNN – were used. In addition, a baseline classifier – kNN – was tested for comparison purposes.

## Results and discussion

The classifier configurations in Table 2 were explored in order to find the ones which optimized performance.

**Table 2:** Explored classifier architectures

Classifier	Parameters	Values
MLPI	number of hidden layers	1
	number of neurons in the hidden layer:	even numbers in [6,28]
MLPII	number of hidden layers	2
	number of neurons:	
	– first hidden layer	even numbers in [6,28]
	– second hidden layer	half the number of neurons of the first layer
LVQNN	number of neurons in the hidden layer	even numbers in [6,28]
RBFNN	spread value	powers of two with integer exponents in [-1,15]
PNN	spread value	powers of two with integer exponents in [-1,15]
k-NN	number of neighbors	integers in [1,15]

The selected sampling strategy was leave-one-out, so as to assure that, in the experiments with the original dataset, there would always be at least one sample of the negative response class in the training set. The classification accuracies regarding the 4 classes were computed. In order to determine the appropriate settings of each classifier, for each experiment, tested configurations were ranked by performance for each class, and the one with the best average rank (over the 4 classes) would be the one selected. The next step was to determine which classifiers were the best for each experiment and the respective performances. For classifier comparison, considering their performances with respect to all classes, Friedman and Bonferri-Dunn [32] tests were performed.

In this work, *N*=4 (classes) and *k*=6 (classifiers). The 6 classifiers were ranked for each of the 4 classes, based on their scores. In the presence of ties, the involved ranks were averaged. The Friedman statistic (*T*1) was computed based on such ranks. By looking at *F* distribution tables, *F*(5,15)=2.90. Table 3 shows the main results of Friedman and Bonferri-Dunn tests for each experiment.

**Table 3:** Classifier accuracies (ranks) for each class, the Friedman statistic (*T*1) and average classifier rank, for the experiments with the 4 datasets

Dataset	Classifier	Negative	Neutral	Positive (partial)	Positive (complete)	Average rank
Original *T*1=1.956	kNN	0 (4.5)	0.167 (5.5)	0.741 (4)	0.893 (4)	–
	LVQNN	0 (4.5)	1.0 (1)	1.0 (1)	0.929 (2)	–
	MLPI	1.0 (1)	0.667 (2)	0.778 (3)	0.893 (4)	–
	MLPII	0.5 (2)	0.5 (3)	0.889 (2)	0.893 (4)	–
	RBFNN	0 (4.5)	0.167 (5.5)	0.444 (6)	0.964 (1)	–
	PNN	0 (4.5)	0.333 (4)	0.704 (5)	0.750 (6)	–
SMOTE *T*1=12	kNN	0.125 (6)	0.417 (6)	0.630 (4.5)	0.893 (4)	5.125
	LVQNN	1.0 (1.5)	1.0 (1)	1.0 (1)	1.0 (1)	1.125
	MLPI	0.5 (3)	0.5 (5)	0.778 (3)	0.930 (2.5)	3.375
	MLPII	1.0 (1.5)	0.917 (2)	0.852 (2)	0.930 (2.5)	2
	RBFNN	0.25 (5)	0.667 (4)	0.444 (6)	0.393 (6)	5.25
	PNN	0.375 (4)	0.889 (3)	0.630 (4.5)	0.679 (5)	4.125
PCA *T*1=4.241	kNN	0 (4.5)	0.167 (5.5)	0.741 (5)	0.893 (3.5)	4.625
	LVQNN	0 (4.5)	1.0 (1)	1.0 (1)	0.929 (1.5)	2
	MLPI	0.5 (1.5)	0.5 (2.5)	0.778 (3.5)	0.929 (1.5)	2.25
	MLPII	0.5 (1.5)	0.5 (2.5)	0.889 (2)	0.893 (3.5)	2.375
	RBFNN	0 (4.5)	0.333 (4)	0.667 (6)	0.464 (6)	5.125
	PNN	0 (4.5)	0.167(5.5)	0.778 (3.5)	0.821 (5)	4.625
SMOTE+PCA *T*1=4.602	KNN	0.5 (3.5)	0.67 (4)	0.630 (5)	0.893 (4)	4.125
	LVQNN	1.0 (1)	1.0 (1)	0.963 (1)	1.0 (1)	1.0
	MLP	0.125 (6)	0.833 (2.5)	0.704 (4)	0.964 (2.5)	3.75
	MLPII	0.5 (3.5)	0.833 (2.5)	0.815 (2)	0.964 (2.5)	2.625
	RBFNN	0.375 (5)	0.417 (6)	0.741 (3)	0.714 (5)	4.75
	PNN	0.625 (2)	0.583 (5)	0.556 (6)	0.679 (6)	4.75

It can be observed that, in the experiment with the original dataset, *T*1<*F*(5,15), so the null hypothesis cannot be rejected, i.e., one cannot state that there is a significant statistical difference among the performances of the 6 classifiers. In the remaining experiments, *T*1>*F*(5,15) – one can state that a significant statistical difference exists among the performances of the 6 classifiers. For these experiments, the Bonferri-Dunn test was performed, with the aim to compare the performances of the different ANNs with the baseline classifier, kNN. The critical value (*CD*) was computed for a 5% significance level – *CD*=1.92. The average ranks of the classifiers were also computed. Only those with an average rank better than that of the baseline classifier by more than the *CD* value can be considered to significantly outperform the baseline classifier.

Looking at Table 3, one can conclude that:

1. In the experiment with the original dataset after SMOTE, LVQNN and MLPII significantly outperform kNN;
2. In the experiment with the original dataset after PCA, LVQNN, MLPI and MLPII significantly outperform kNN;
3. In the experiment with the original dataset after SMOTE+PCA, LVQNN is the only classifier to significantly outperform kNN.

Thus, in 3 out of 4 scenarios it was verified that the selection of a more complex classifier than kNN, such as LVQNN, MLPI or MLPII, pays off in terms of performance. In the experiment with the original dataset, such selection seems to be unjustified.

Taking a closer look at performance itself, one can draw a few relevant conclusions:

1. The considered set of features allows for very high classification performances, when data is properly balanced;
2. In data imbalancement scenarios, performance is clearly poorer – the introduction of SMOTE markedly improves performance;
3. Dimensionality reduction to two components using PCA does not seem to have significant effects on performance. As such, dimensionality reduction is advantageous for us, as it allows for a serious reduction of computational load while preserving performance.

As for time complexity, although it is not critical in this project, as real-time is not required, an idea of its order with respect to each of the adopted classifiers can be provided: the average kNN ran in 57 seconds, RBFNN and PNN in the 1 hour range (1.4 h and 0.7 h), LVQNN and the MLPs (the highest performing classifiers) ran in the 14, 14 and 20 hours respectively. It is also important to note that for the PCA experiments, these times are reduced drastically for the seconds and minutes range for all the algorithms.

## Conclusions

The analysis of the radiotracer fixation in PET/CT is a rather complex task for nuclear clinicians, in their daily practice. This issue is even more critical as worldwide-accepted measures (e.g. SUV), that could constitute an important bridge to reduce such complexity, can be largely biased as result of multiple factors. However, the successful automated analysis of such images, for instance in the context of treatment response, could result in an increase of the patient survival especially in oncological realities.

In this project, an approach to automatically evaluate oncological treatment response using PET/CT images was proposed. To achieve that, more than 300 features were collected and different types of neural networks were implemented. These features combine clinical information such as patient weight or lesion SUVmax with a series of image features including mainly textural information.

The results show that the considered set of features allows for the achievement of very high classification performances, especially when data is properly balanced. After synthetic data generation and PCA-based dimensionality reduction to only two components, LVQNN assured classification accuracies of 100%, 100%, 96.3% and 100% regarding the 4 response-to-treatment classes.

Regarding future directions, the next stage will be applying evolutionary approaches for obtaining a reliable evaluation function of treatment response based on the features collected in this work. Moreover, the intention is to extend these works to other oncological diseases.

## Abbreviations

ANN: artificial neural network
AUC: Area under the curve
CD: Critical value
CT: Computed tomography
FDG: Fluoro-deoxy glucose
HGD: Histograms of gradient divergence
ICA: Independent component analysis
KDD: Knowledge discovery in databases
kNN: k nearest neighbors
LBP: Local binary patterns
LVQNN: Learning vector quantization neural network
MLP: Multi layer perceptron
PET: Positron emission tomography
PNN: Probabilistic neural network
RBFNN: Radial basis function neural network
ROI: Region of interest
SIFT: Scale invariant transform
SMOTE: Synthetic minority over-sampling technique
SUV: Standardized uptake value
